# Correction to “Association between the Stanford Integrated Psychosocial Assessment for Transplant and 1‐year medication nonadherence after living kidney transplantation”

**DOI:** 10.1002/pcn5.70152

**Published:** 2025-07-28

Takano K, Kobayashi S, Oshibuchi H, Tsutsui J, Ito S, Kamba R, et al. Association between the Stanford Integrated Psychosocial Assessment for Transplant and 1‐year medication nonadherence after living kidney transplantation. Psychiatry Clin Neurosci Rep. 2025; 4:e70128. https://doi.org/10.1002/pcn5.70128.

The authors have identified a copyright infringement in Table 2 in the article.

**Table 2:** 

	BAASIS^©^ items (*N* = 111)	*N*	(%)
**1A.**	Taking nonadherence		
	Yes	13	(11.7)
	No	98	(88.3)
	Once	9	(8.1)
	Twice	2	(1.8)
	Three times	1	(0.9)
	Four times	0	(0)
	More than 4 times	1	(0.9)
**1B.**	Drug holidays		
	Yes	0	
	No	13	
**2**	Timing non‐adherence		
	Yes	38	(34.2)
	No	73	(65.8)
	Once	14	(12.6)
	Two to three times	12	(10.8)
	Four to five times	6	(5.4)
	Every 2 to 3 days	6	(5.4)
	Almost every day	1	(0.9)
**3**	Dose alteration		
	Yes	0	(0)
	No	111	(100)
**4**	Discontinuation		
	Yes	0	(0)
	No	111	(100)
**5**	Non‐initiation		
	Yes	1	
	No	40	
	**Total number categorized as showing nonadherence**	44	(39.6)

Abbreviation: BAASIS^©^, the Basel Assessment of Adherence to Immunosuppressive Medication Scale^©^.

The table presents the full scale of the BAASIS^©^ (Basel Assessment of Adherence to Immunosuppressive Medications Scale), which the authors had included in error and is a violation of the BAASIS^©^ copyright terms.

Below is the revised Table 2, which adheres to the BAASIS^©^ copyright by providing information on the scale's use and scoring without reproducing the copyrighted items verbatim.

The table has also been corrected in the article itself.

The authors apologize for this error.

